# Supplementary material for: Thermally stable threshold selector based on CuAg alloy for energy-efficient memory and neuromorphic computing applications
Source: Nat Commun. 2023 Jun 6;14:3285. doi: 10.1038/s41467-023-39033-z (PMC10244361; doi:10.1038/s41467-023-39033-z)
Supplement: Supplementary file 1 — Supplementary Information [file 41467_2023_39033_MOESM1_ESM.pdf]

Supplementary Information

Thermally-stable threshold selector based on CuAg alloy for energy-efficient memory and neuromorphic computing applications

Xi Zhou, Liang Zhao*, Chu Yan, Weili Zhen, Yinyue Lin, Le Li, Guanlin Du, Linfeng Lu, Shan-Ting Zhang, Zhichao Lu, Dongdong Li*

*email: lzhao2020@zju.edu.cn, lidd@sari.ac.cn

This file includes:

Supplementary Figures 1–18

Supplementary Tables 1–2

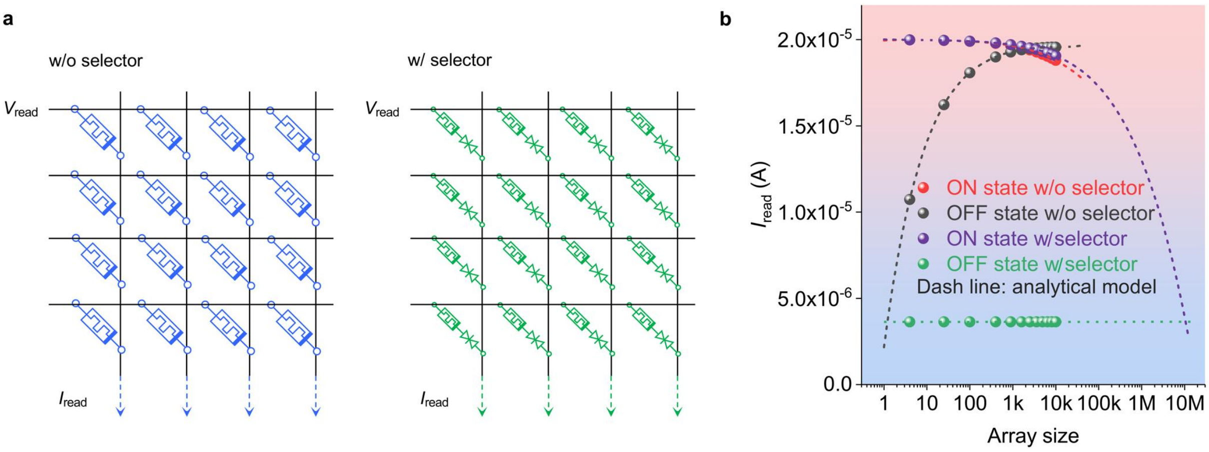

Supplementary Figure 1. a, Schematic diagram of the worst-case read current (I_{read}) of the cross-point array simulated using SPICE (see Methods). The worst-case of the ON state is to read the cell that is farthest from the power line, and then the other cells are OFF, and the worst-case of the OFF state is to read the cell that is closest to the power line, and the other cells are ON. **b**, I_{read} variation with array size with and without selector, where the spheres indicate the results of simulations using SPICE and the dashed lines indicate the results of simulations using the analytical model. The simulation parameters are shown in Supplementary Table **1**. Without the selector, it is no longer possible to perform a correct read operation the array size reaches 10 (worst case ON/OFF ratio<2), while the case with the selector is able to allow a larger integration (>1 M) and operation window.

Supplementary Table 1. Device parameters for the simulation process include read voltage (V_{read}), ON and OFF state resistance (R_{ON} and R_{OFF}) and series resistance (R_{sr}).

Parameter name	Value
R_{ON}	10000 Ω
R_{OFF} (w/o selector)	200000 Ω
R_{OFF} (w/ selector)	10^{11} Ω
R_{sr} (between cells)	5 Ω
R_{sr} (output terminal)	100 Ω
V_{read}	0.4 V

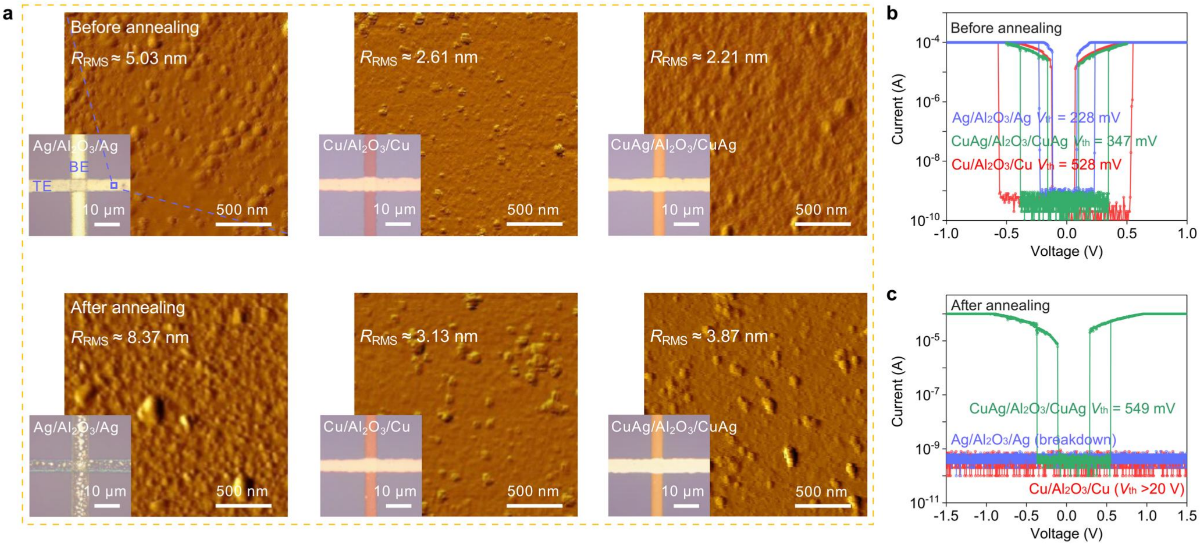

Supplementary Figure 2. a, Surface morphologies of Ag/Al_2O_3/Ag, Cu/Al_2O_3/Cu and CuAg/Al_2O_3/CuAg devices before and after annealing in Ar atmosphere at 400 ^{o}C for 1 hour. **b**, *I*–*V* characteristics of Ag/Al_2O_3/Ag, Cu/Al_2O_3/Cu and CuAg/Al_2O_3/CuAg selectors before annealing. **c**, *I*–*V* characteristics of annealed Ag/Al_2O_3/Ag, Cu/Al_2O_3/Cu and CuAg/Al_2O_3/CuAg selectors.

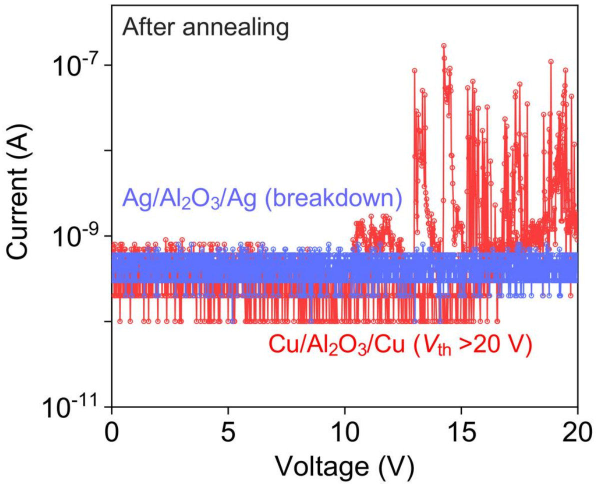

Supplementary Figure 3. *I*–*V* characteristics of annealed Ag/Al_2O_3/Ag and Cu/Al_2O_3/Cu selectors (400 °C, Ar atmosphere, 1 hour).

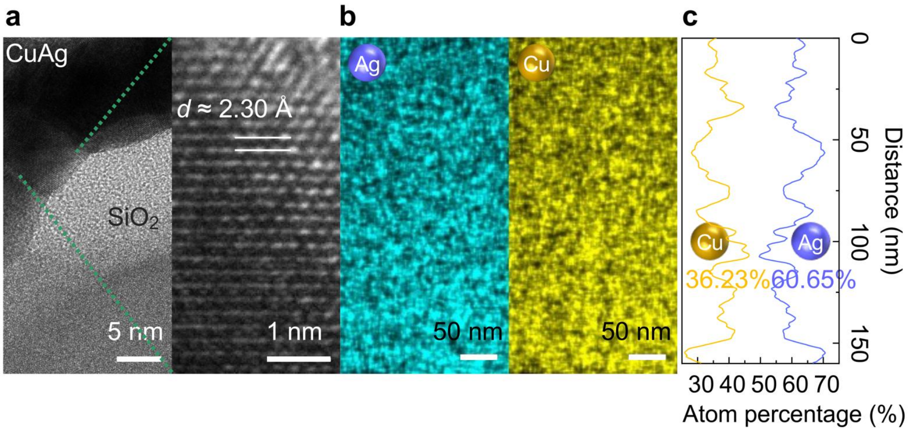

Supplementary Figure 4. a, STEM image of the CuAg electrode. The lattice fringe with an interplanar spacing (*d*) of 2.30 Å is attributed to the CuAg alloy (111) plane, which is consistent with XRD results (Fig. 1**f**). As for XRD results, The (111) interplanar spacings (d_{111}) of Ag, Cu and CuAg, calculated by the Bragg's Law[1] from XRD patterns, are 2.35, 2.08 and 2.30 Å, respectively. **b**, The EDS mapping and (**c**) linear sweep results for Ag and Cu elements in CuAg electrode. The mean values of the atomic percentages of Cu and Ag are 36.23% and 60.65%, corresponding to a Cu/Ag atomic ratio of about 3:5. The higher Ag content supports why the d_{111} of the CuAg alloy is closer to that of Ag. The CuAg prepared by co-sputtering is determined by XRD as a nanocrystalline alloy (Fig. 1**f**). The diffraction peak at 39.06^{o} is significantly broadened with a full width at half maximum (~1.34^{o}). The corresponding crystalline size (*D*) is estimated to be ~6.29 nm acquired by Debye-Scherrer equation[2].

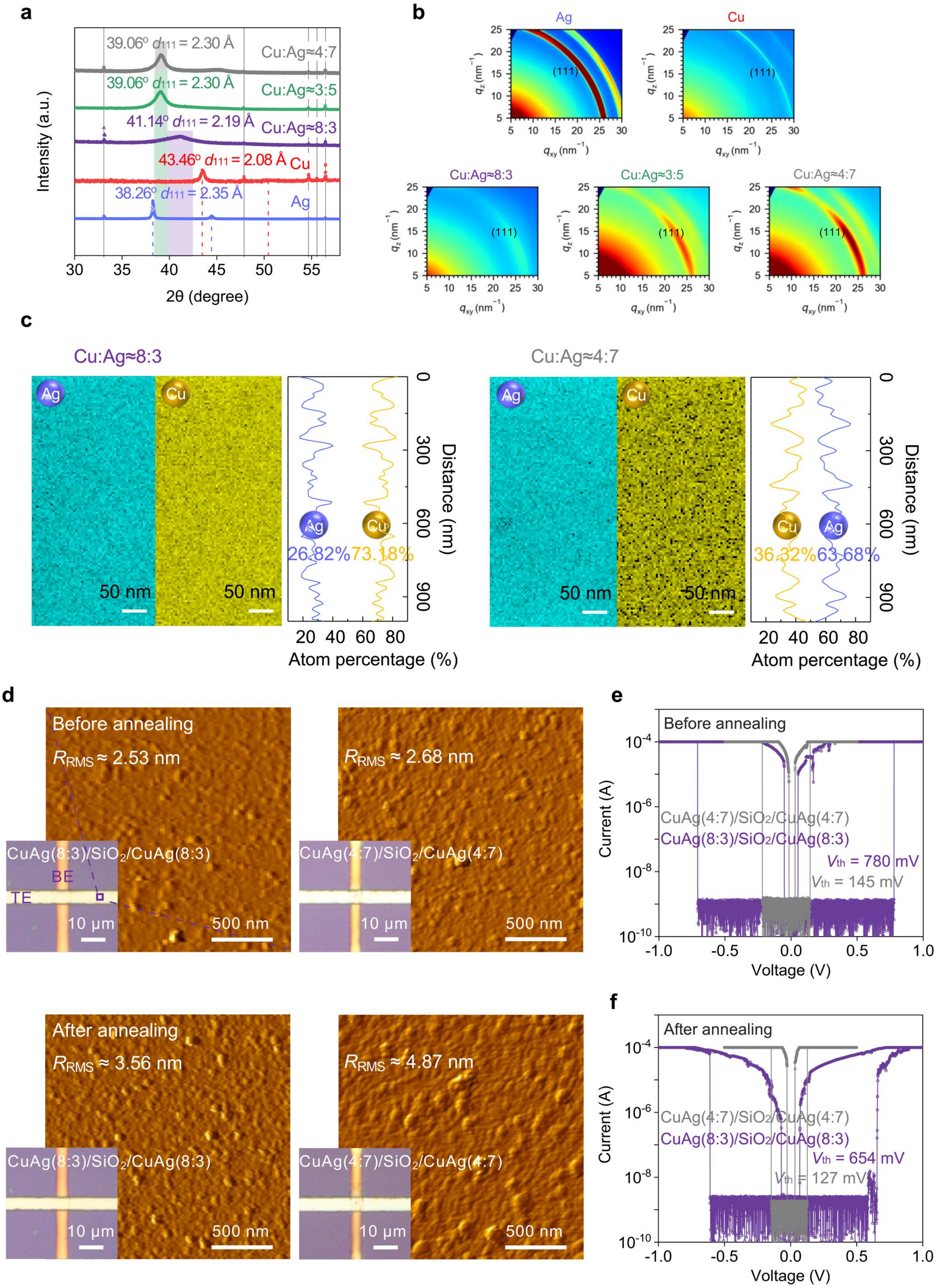

Supplementary Figure 5. Crystal structures of Cu, Ag, and three copper-silver alloy films determined by **a,** XRD and **b,** GIXRD. **c,** The compositions of the two additional CuAg alloy films determined by EDS. **d,** Surface morphologies of CuAg(8:3)/SiO_2/CuAg(8:3) and

$CuAg(4:7)/SiO_2/CuAg(4:7)$ devices before and after annealing in Ar atmosphere at 400 °C for 1 hour. **e,** *I–V* characteristics of $CuAg(8:3)/SiO_2/CuAg(8:3)$ and $CuAg(4:7)/SiO_2/CuAg(4:7)$ devices before annealing. **f,** *I–V* characteristics of annealed $CuAg(8:3)/SiO_2/CuAg(8:3)$ and $CuAg(4:7)/SiO_2/CuAg(4:7)$ devices. Combining the results of all devices, the effect of Cu/Ag ratio on thermal stability can be summarized as follows: pure Ag is very unstable, and due to its soft texture and agglomeration phenomenon, split Ag islands will form after high temperature, which is not compatible with CMOS processes. The Cu doping in Ag can stabilize the threshold switching phenomenon over a wide range of doping concentration, i.e. threshold switching can be maintained after annealing at 400 °C. However, when the ratio of Cu is high (e.g., CuAg(8:3)), the device exhibits a potential oxidation phenomenon that causes an increase in the ON state resistance. Such a phenomenon and the large threshold voltage (V_{th}) increase after annealing of the pure Cu selectors can be explained by the Cu oxidation mechanism. Also, the post-annealing changes of ON state resistance and V_{th} at higher Cu concentrations are not fully controllable. This suggests that electrode materials with higher Ag content are preferred (e.g. Cu/Ag between 4:7 and 3:5).

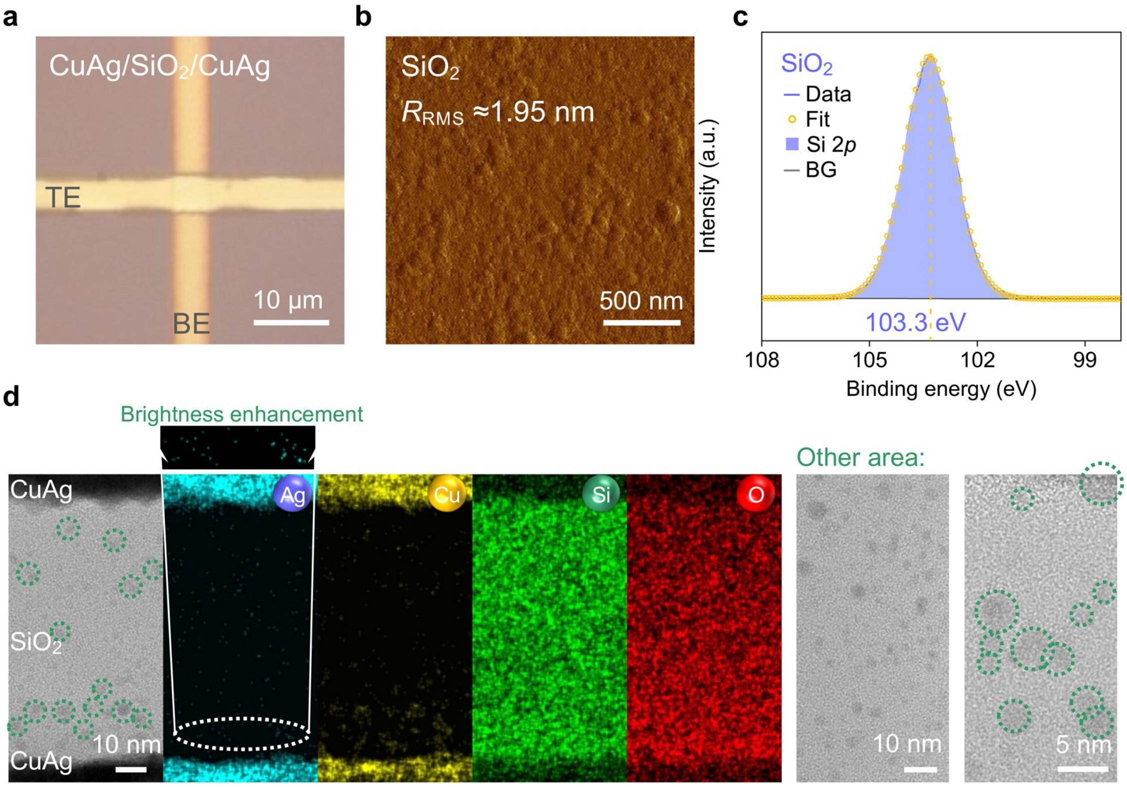

Supplementary Figure 6. Structure of the CuAg/SiO_2/CuAg selector and some basic properties of the SiO_2 interlayer. a, A top view of CuAg/SiO_2/CuAg selector, the electrode linewidths of bottom electrode (BE) and top electrode (TE) are 5 μm. Since the SiO_2 layer and the TE are patterned together, the SiO_2 cannot be directly observed. **b**, AFM image of the SiO_2 film. **c**, XPS spectrum of the SiO_2 film. **d**, Cross-sectional STEM image of the as-fabricated CuAg/SiO_2/CuAg selector and corresponding EDS elemental mapping of Ag, Cu, Si, and O. The green circles mark the diffused metallic spherical particles, which appear spherical due to minimized interfacial energy[3]. The other SiO_2 cross section (named: other area) with more pronounced metal particles, where the metal spheres are between 1 and 5 nm in diameter. These clusters may contribute to the partitioning of a thick SiO_2 layer into thin regions with a low migration barrier for metal ion transfer among the clusters, thus rendering the electroforming-free behaviors. We speculate that the cause for this type of behaviors is due to the electron beam evaporation process used to deposit SiO_2, which may result in a more porous film. This combined with the low migration barriers of Ag and Cu in SiO_2 (Ag:

0.61 eV (see Fig. S10); Cu: 0.4~1.1 eV[4]) may contribute to the electroforming-free selector even with thick SiO_2. An alternative ALD method is expected to significantly reduce the required SiO_2 thickness.

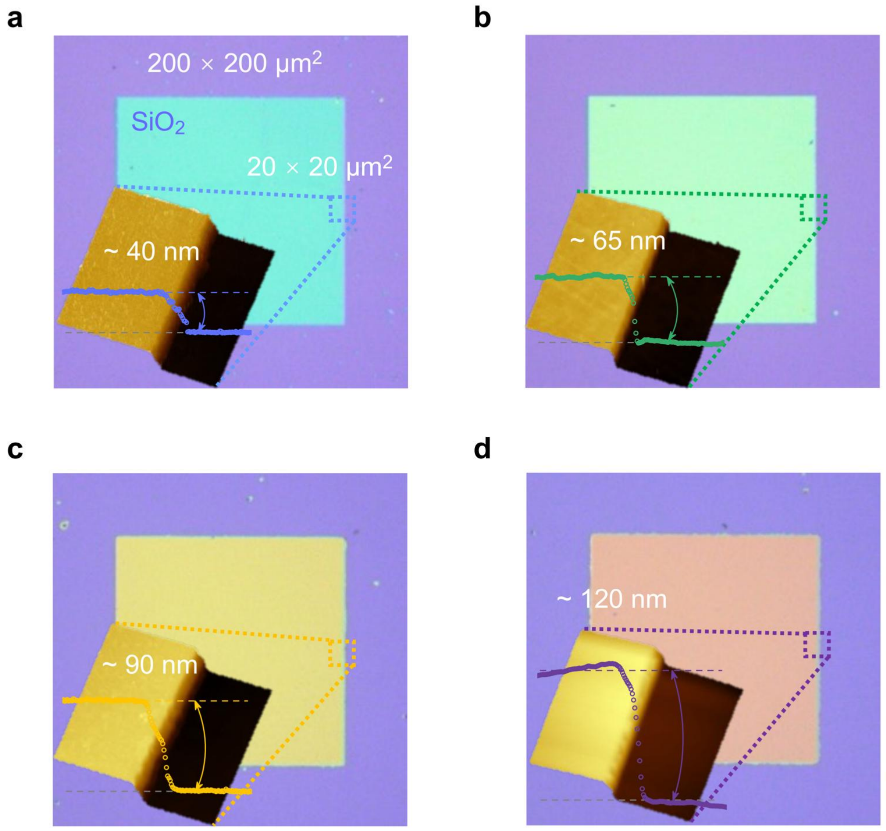

Supplementary Figure 7. Calibration of the thickness of the SiO_2 interlayers for CuAg/SiO_2/CuAg selectors. The SiO_2 interlayers are patterned and tested using AFM to obtain their thicknesses of (**a**) 40, (**b**) 65, (**c**) 90, and (**d**) 120 nm, respectively.

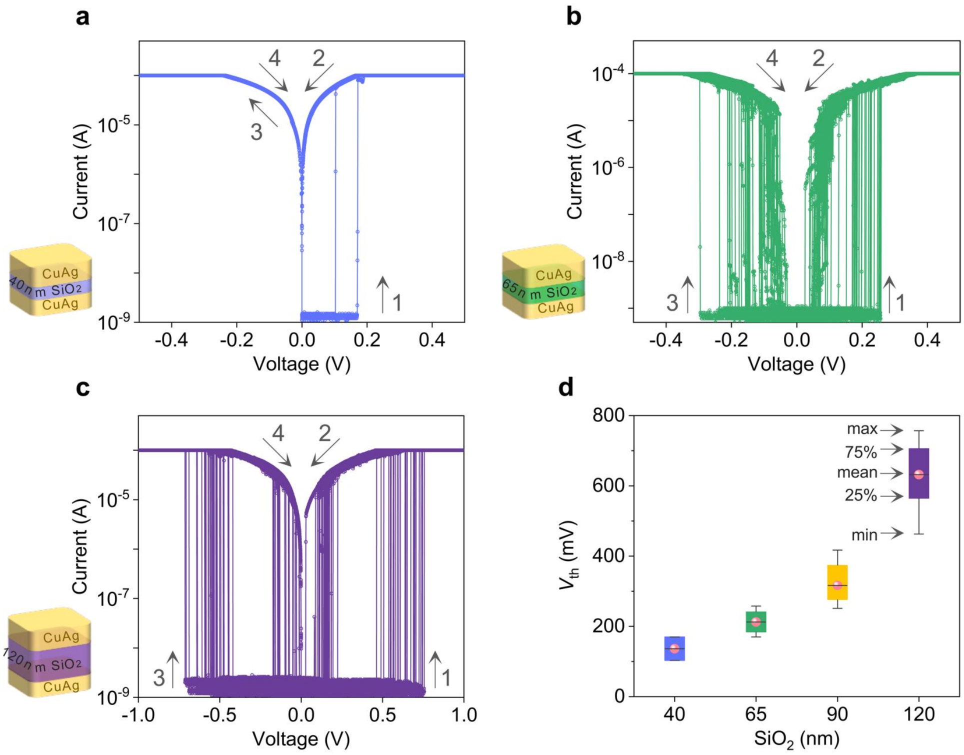

Supplementary Figure 8. Adjustable threshold voltage (V_{th}) achieved by varying the thickness of the SiO_2 dielectrics. *I–V* characteristics of (**a**) CuAg/40 nm SiO_2/CuAg, (**b**) CuAg/65 nm SiO_2/CuAg and (**c**) CuAg/120 nm SiO_2/CuAg selectors. **d**, Variation of the V_{th} as a function of SiO_2 interlayer thickness. For selectors with 40 nm SiO_2, it is difficult to maintain the symmetric threshold switching characteristic as they remain in the ON state after the first turn-on and are difficult to return to the OFF state probably due to the presence of pin-holes. While the devices with 65 nm SiO_2 demonstrate the complete ON/OFF switching process with an average V_{th} of 211 mV (Standard deviation $\sigma \approx 30$ mV). Devices with a SiO_2 interlayer thickness of 120 nm have a higher average V_{th} of 631 mV ($\sigma \approx 84$ mV). These results confirm that the V_{th} of proposed selectors can be tuned by adjusting the interlayer thickness.

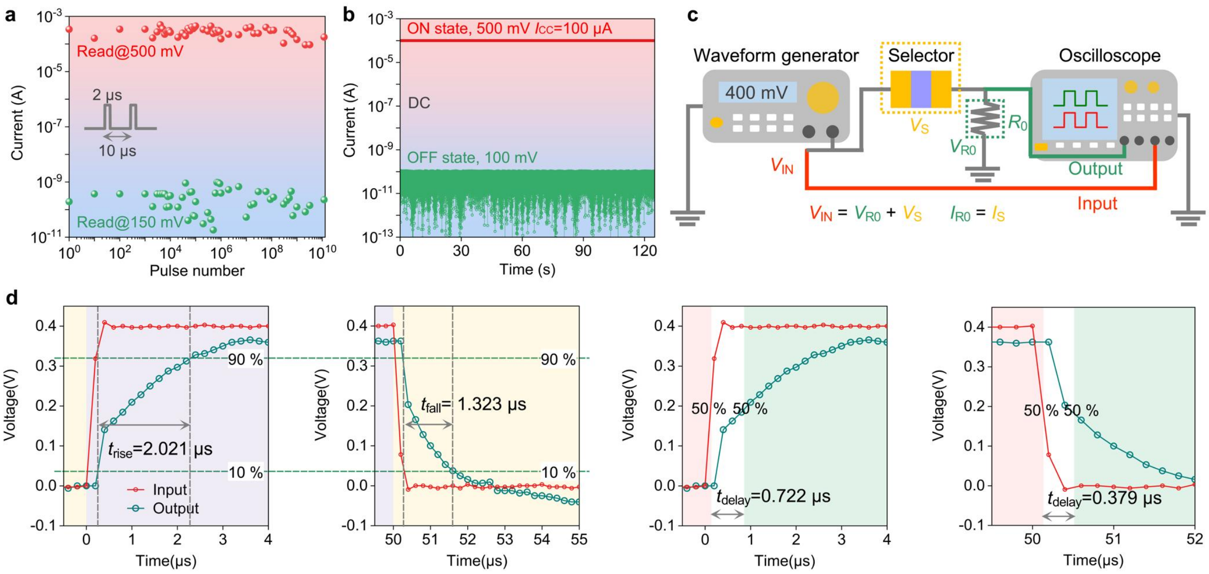

Supplementary Figure 9. Endurance and temporal behaviors of the CuAg/SiO_2/CuAg selector. a, Pulse endurance and **b,** DC retention of the CuAg/90 nm SiO_2/CuAg selector. The CuAg/SiO_2/CuAg selector shows good endurance under AC and DC voltage stimulations, capable of maintaining threshold switching after >10^{10} AC pulses or more than 120 seconds of DC stimulation. **c,** Schematic diagram of the rise, fall and turn-on/turn-off delay times measurement setup. The waveform input from the waveform generator (Keysight 33250A) and the waveform output from the measurement setup are measured by an oscilloscope (Agilent MSO7054A). **d,** Rise, fall and turn-on/turn-off delay times of the CuAg/90 nm SiO_2/CuAg selector. The relevant parameters of the devices are only conservatively evaluated due to the circuit structure and test accuracy. Consider the RC delays associated with top and bottom electrodes, we expect the actual switching delay time to be much faster than the obtained results, if the test structure can be optimized.

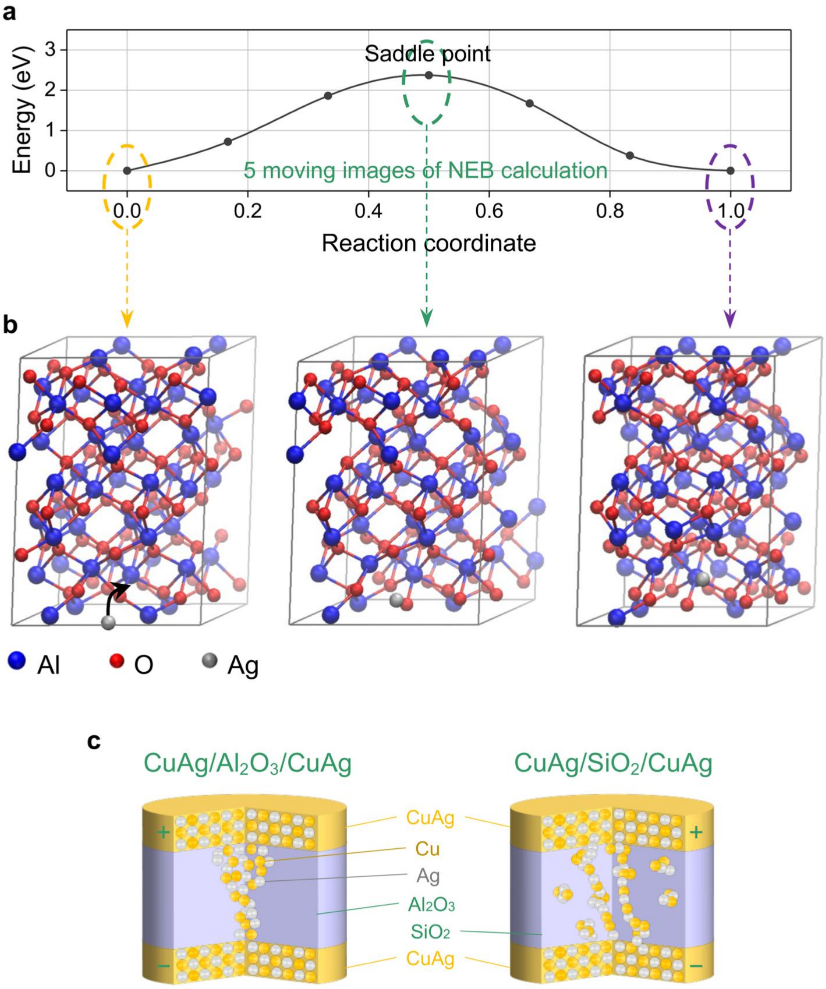

Supplementary Figure 10. a, Energy profile of Ag migration in Al_2O_3, calculated by density functional theory (DFT) and the nudged-elastic-band (NEB) method. The simulated system comprises of a supercell of trigonal Al_2O_3 ($R\bar{3}c$ space group) with 48 Al atoms and 72 O atoms. The DFT simulations are carried out based on the projector augmented-wave (PAW) method[5,6] and the general-gradient approximation (GGA) with Perdew–Burke–Ernzerhof (PBE)[7] pseudopotentials, as implemented by the Vienna Ab initio Simulation Package (VASP)[8–11]. The initial and final configurations with one additional Ag atom introduced as an interstitial are fully relaxed to obtain the ground state, with electronic relaxation converged to 1×10^{-8} eV/atom and interatomic forces smaller than 1×10^{-4} eV/Å. The relaxed unit cells are then used to construct the NEB calculations with the climbing image method (CI-NEB)[12–14], which allows more accurate finding of saddle points with fewer images (5 images in this case).

Multiple potential migration paths are calculated and the one with lowest energy is plotted. **b**, The Al_2O_3 supercell structures along the Ag migration path corresponding to the migration barrier in (**a**), relaxed with the CI-NEB. The Supplementary Table **2** summarizes the migration barriers and Bader charges for Ag/Ag^+ in SiO_2, ZrO_2 and Al_2O_3. The results for SiO_2 and ZrO_2 are adapted from the ref. [15]. In ZrO_2, because the Bader charges of Ag and Ag^+ are 0.57 *e* and 0.58 *e*, and the valence state of Zr is variable, thus the Ag interstitial always stays as a cation[14]. In SiO_2 and Al_2O_3, the Bader charges of Ag (0.16 *e* and 0.066 *e*) are substantially lower than Ag^+ (0.72 *e* and 0.327 *e*), thus there are two states (Ag and Ag^+) for the silver interstitial. On the other hand, the migration barrier results show that the barrier of Ag and Ag^+ in Al_2O_3 (2.37 eV and 3.47 eV) is much larger than that in SiO_2 (0.61 eV and 0.53 eV), indicating that the formation of conductive filaments in SiO_2 is easier. In general, the Ag^+ in ZrO_2 migrates continuously with the electric field and is easy to form non-volatile memristors[16]. The presence of both Ag and Ag^+ in Al_2O_3 and SiO_2 makes it easy to form volatile selectors through clustering. At the same time, the migration barrier of silver in SiO_2 is much lower, thus the switching speed is faster and it is easier to form electroforming-free devices. Combined with the results of STEM (Fig. S4**d**), metals exist in the form of clusters in SiO_2. When a bias voltage is applied, the clusters on the anode side release metals, and the clusters on the cathode side accumulate metals until conductive filaments are formed connecting both terminals. When the applied voltage is less than the hold voltage, the metal in the filament migrates back into the clusters or electrodes due to the very low migration barrier in SiO_2, enabling volatile resistance switching. **c**, Schematic diagram of the conductive filament morphology of CuAg/Al_2O_3/CuAg and CuAg/SiO_2/CuAg selectors in the ON state. For the CuAg/Al_2O_3/CuAg selector, even though the migration barrier of metal atoms in Al_2O_3 is large, conical conductive filaments can still be formed in the ultrathin Al_2O_3 film. For the CuAg/SiO_2/CuAg selector, due to the small migration barrier of the metal, in the ON state, the metal in the electrodes and clusters form connected conductive filaments.

Supplementary Table 2. The migration barriers and Bader charges for Ag/Ag^+ in SiO_2, ZrO_2 and Al_2O_3. The results for SiO_2 and ZrO_2 are adapted from the ref. [15].

Dielectrics	Migration barrier (eV)		Bader charges	
	Ag	Ag^+	Ag (*e*)	Ag^+ (*e*)
SiO_2	0.61	0.53	0.16	0.72
ZrO_2	2.39	2.48	0.57	0.58
Al_2O_3	2.37	3.47	0.066	0.327

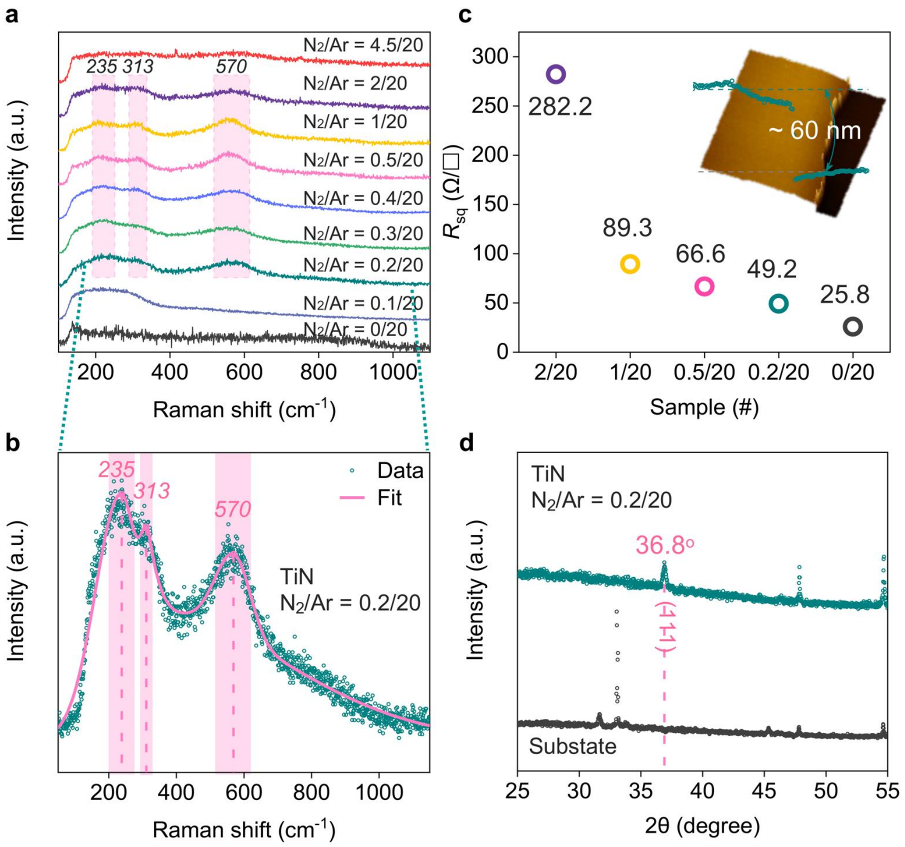

Supplementary Figure 11. Basic characteristics about the TiN electrodes. a, Raman spectra of the films prepared by sputtering Ti target in different N_2/Ar flow ratios at room temperature. Controlling the N_2/Ar flow ratio between 0.2/20 and 2/20 enables the TiN films to be obtained. **b**, A close view of Raman spectrum of the TiN film with a N_2/Ar flow ratio of 0.2/20. A series peaks (*235*, *313*, *570* cm^{-1}) corresponding to the characteristic vibration modes of TiN[17]. **c**, Square resistance (R_{sq}) of the TiN films deposited at various N_2/Ar flow ratio. The square resistance of the TiN films decreases as the nitrogen concentration decreases[18]. The thickness of the TiN TE used in the Pt/SiO_2/TiN memristor is ~60 nm with the R_{sq} of ~49.2 Ω/□ (N_2/Ar flow ratio = 0.2/20). **d**, XRD patterns of the SiO_2/Si substrate and TiN film (N_2/Ar flow ratio = 0.2/20). The peak at 36.80° corresponds to the (111) crystal plane of TiN[19].

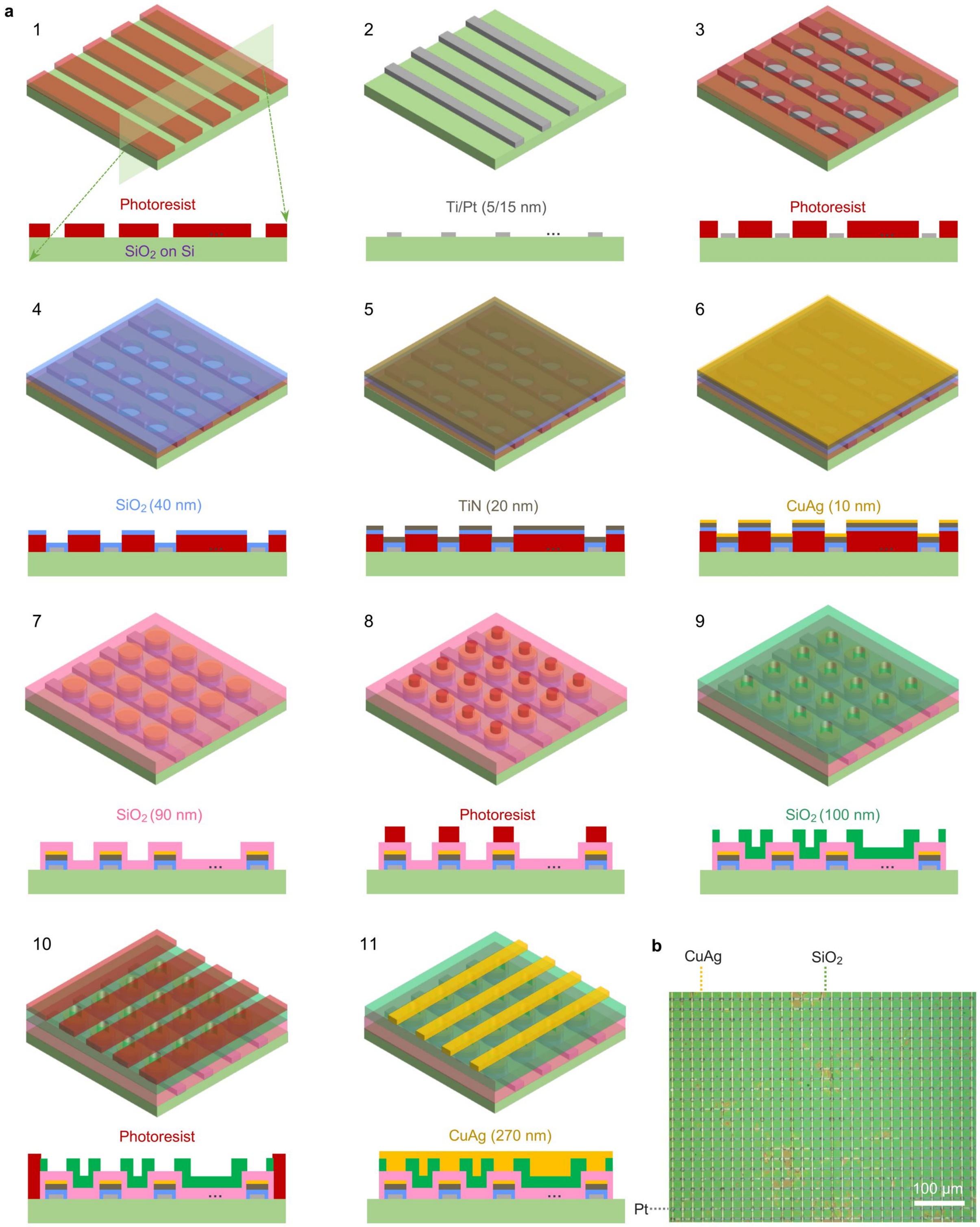

Supplementary Figure 12. A demonstration of 3D stacking 1S1R. a, Schematic top views and side cross-section views of the manufacturing processes of the 1S1R array (some lift-off steps are omitted). After photolithographic patterning (step **1**), electron beam evaporation (step **2**) and lift-off, 64 rows Ti (5 nm)/Pt (15 nm) BE with a linewidth of 2 μm are patterned. Then 64 × 64 sets of circular patterns with 5 μm diameter are obtained by photolithography

(step **3**). In steps **4–6**, SiO_2, TiN, and CuAg are deposited (thicknesses of 40, 20, and 10 nm, respectively), yielding cylinders with diameter of 5 μm after lift-off. The SiO_2 in step **7** is used for the dielectric layer of the selector (thickness = 90 nm), while the SiO_2 (thickness = 100 nm) obtained after steps **8** (photolithography) and **9** (electron beam evaporation) is used to encapsulate the area beyond the cross-point and prevent contact between the TE (CuAg, step **11**) and the intermediate electrode (TiN and CuAg, steps **5** and **6**). The step **9** will leave holes with a diameter of 2 μm. Finally, after steps **10** (photolithography), **11** (co-sputtering) and lift-off, 64 columns CuAg electrodes (thickness = 270 nm) with a linewidth of 2 μm are patterned. At this point, the CuAg TE make contact with the 90 nm SiO_2 layer (step **7**) through the holes left by the 100 nm SiO_2 (step **9**). **b**, A top view of the 64 × 64 1S1R array, the electrode linewidths of BE and TE are 2 μm.

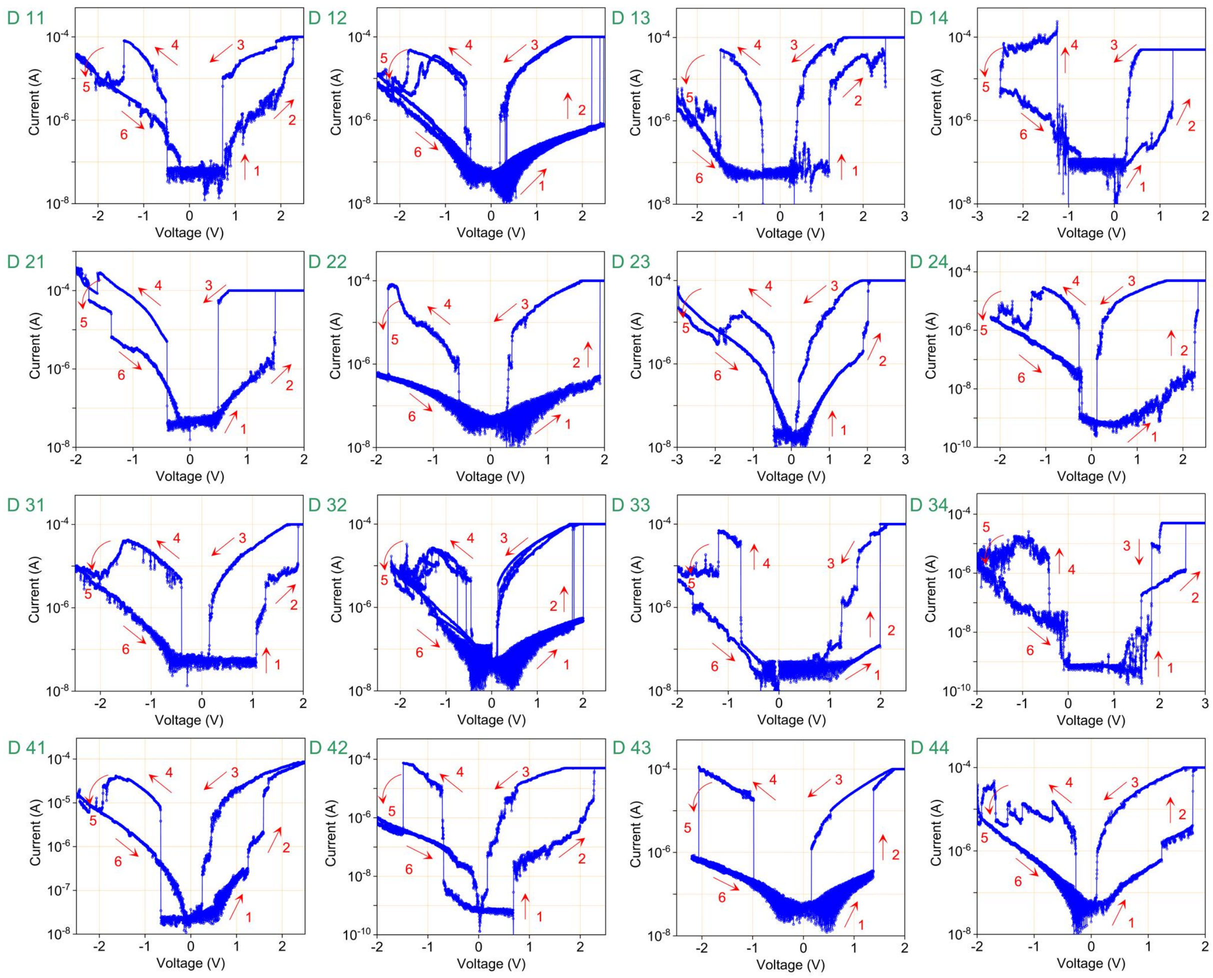

Supplementary Figure 13. *I–V* characteristics of 4 × 4 subarray in a 64 × 64 1S1R array. By summarizing the results of all experiments, the key to influence the yield of 1S1R array is the control of the bottom and middle layer flatness.

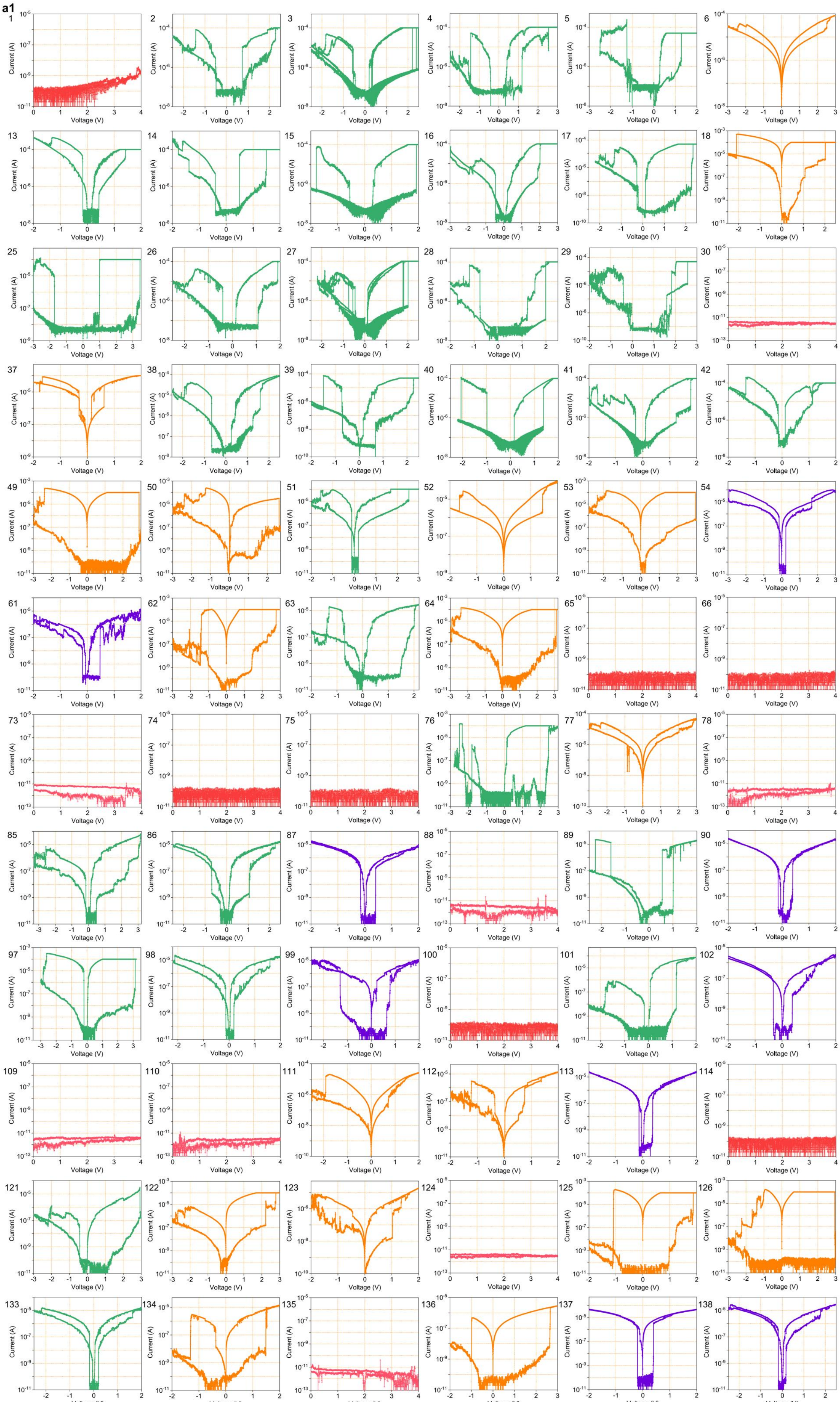

a1
Current (A)
Voltage (V)

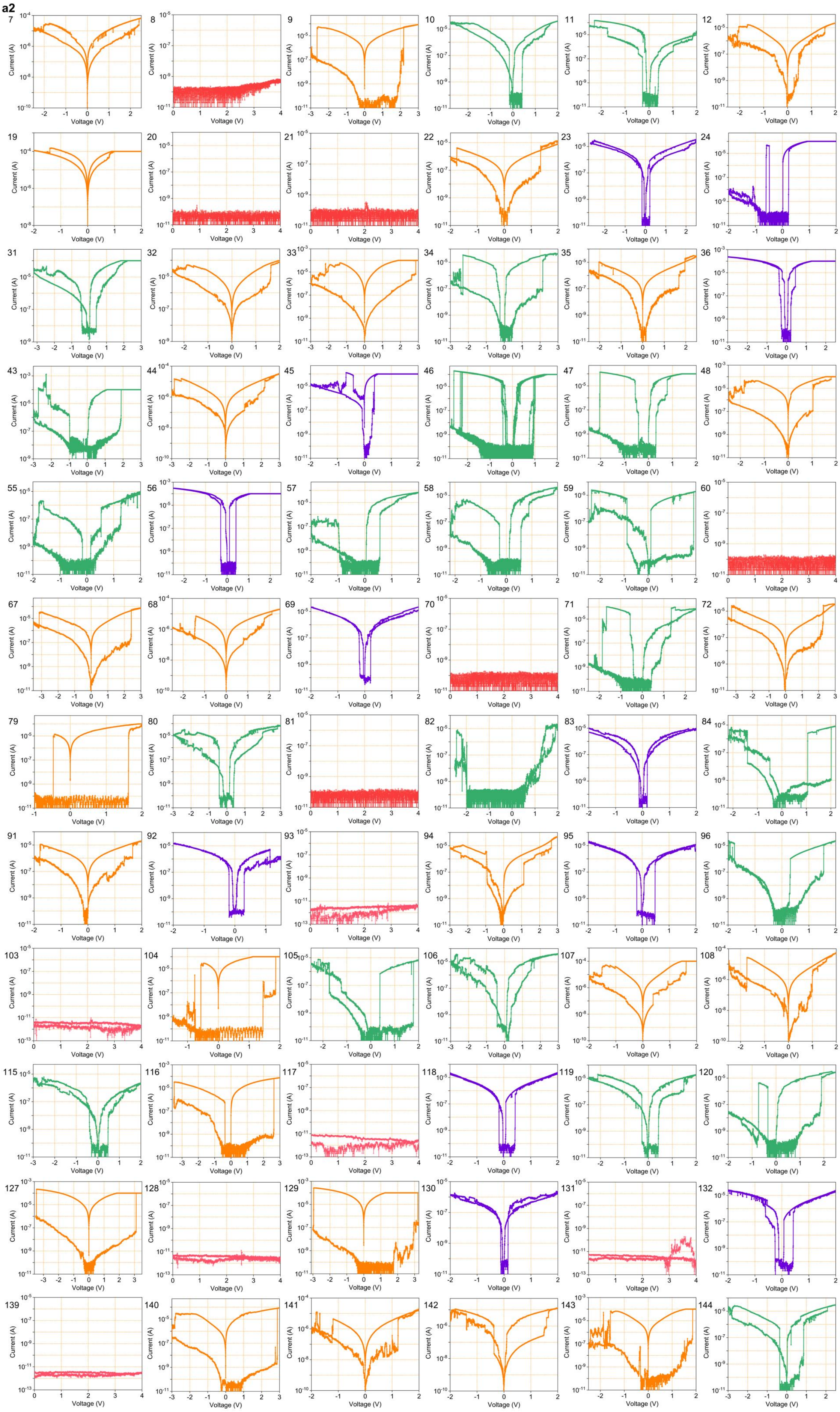
a2
7
8
9
10
11
12
19
20
21
22
23
24
31
32
33
34
35
36
43
44
45
46
47
48
55
56
57
58
59
60
67
68
69
70
71
72
79
80
81
82
83
84
91
92
93
94
95
96
103
104
105
106
107
108
115
116
117
118
119
120
127
128
129
130
131
132
139
140
141
142
143
144
Current (A)
Voltage (V)

b

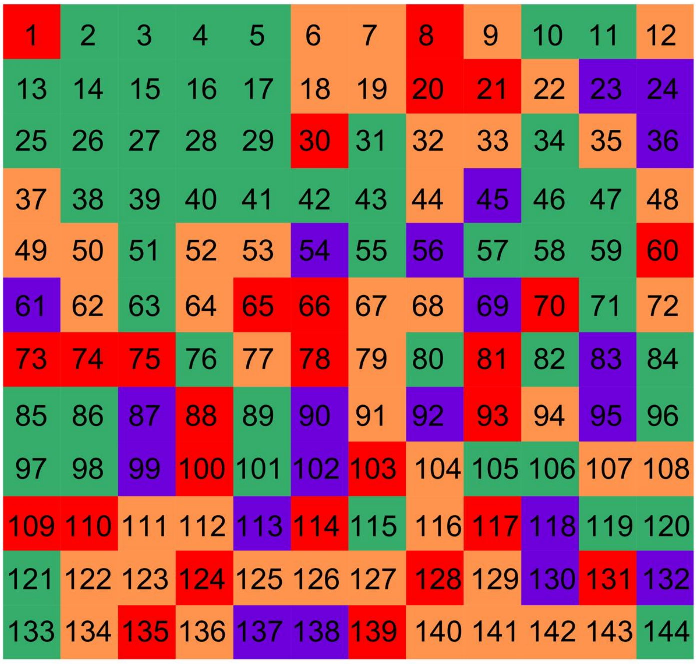

Supplementary Figure 14. Exploration of the yield of the 1S1R array. a1, a2, The electrical properties of 144 devices in the 64 × 64 1S1R array. Green curves: 52 1S1R devices with complete 6 switching processes; purple curves: 21 devices with only normal operation of the selector and shorted memristors; orange curves: 44 devices with only normal operation of the memristors and shorted selectors; red curves: 27 devices with higher operating voltage and presenting open circuits. **b,** The bit map corresponding to 144 devices. For those open devices in the 64 × 64 array, the leakage current is as low as 10^{-11}~10^{-12} A (instrument limit), indicating that the selectors have successfully suppressed the sneak-path leakage currents in a very large array. Thus, in total, there are 52 valid 1S1R devices out of 144 devices, with a yield of 36%.

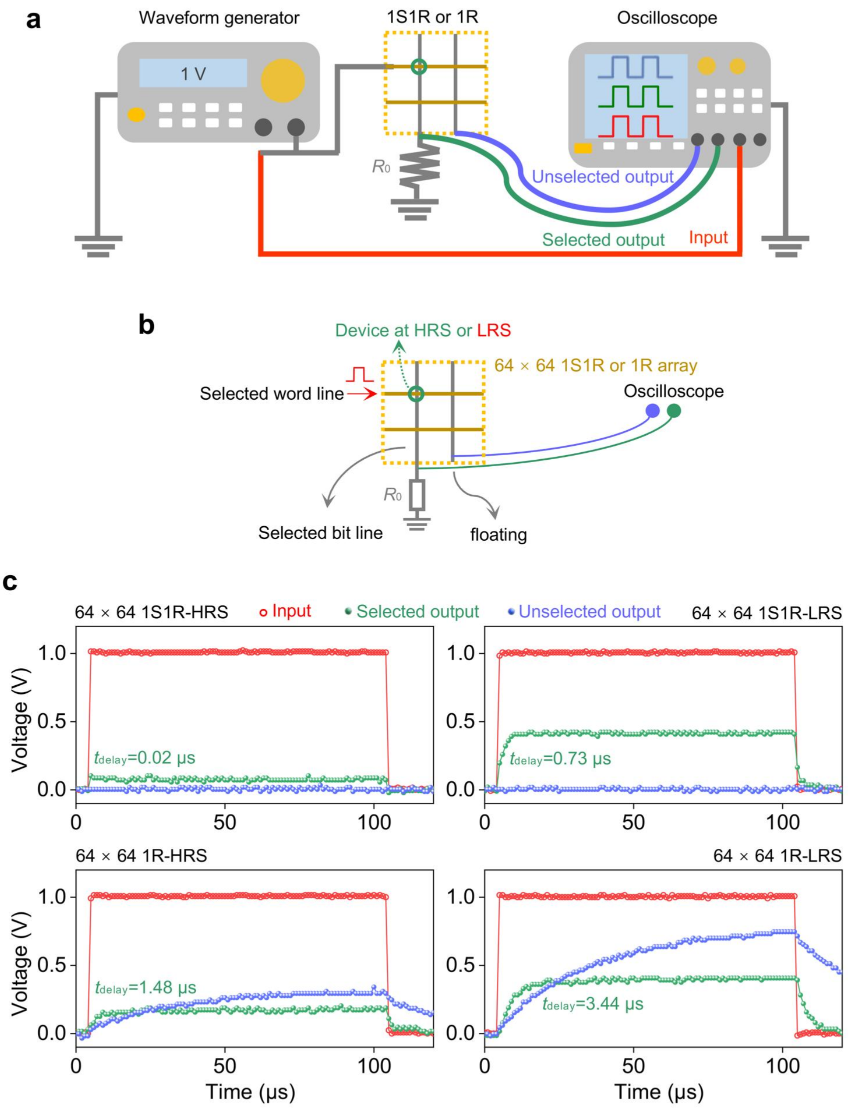

Supplementary Figure 15. Verification circuits of SNN operations (forward propagation of pulses) with 1S1R/1R array as synapses. a, Schematic diagram of the verification circuit. Use a pulse of amplitude 1 V (Keysight 33250A waveform generator) to input to the word line of the selected device and simultaneously read the selected device bit line and nearest bit line with an Agilent MSO7054A oscilloscope. For simplicity, a voltage-dividing resistor R_0 (10 kΩ, approximately equal to the LRS resistor) is connected to the selected output line (bit line) and ground. **b,** Schematic diagram of read operation for selected devices in 64 × 64 1S1R and 1R arrays. **c,** Output results of selected bit line and nearest unselected bit line in 64 × 64 1S1R array and 1R array under a pulse input of 1 V amplitude.

From the results, our discoveries are as follows:

(1) For 64 × 64 1S1R array, the multiplication result of the input voltage versus the weight in terms of RRAM conductance is clearly reflected in the output. Compared to the HRS case, LRS can pass higher current/voltage to the output. Moreover, the unwanted disturbance to unselected output lines is negligible thanks to the presence of selectors which suppress sneak-path currents. The output signal's delay time with reference to the input signal (at 50% voltage range) is 0.02 and 0.73 μs for HRS and LRS, respectively.

(2) For 64 × 64 1R array, the signal on the output line exhibits a longer rise and fall time. The HRS and LRS output delay time is 1.48 and 3.44 μs, respectively. This suggests stronger parasitic effects during the propagation of pulse signal. Moreover, the voltage on the unselected output line also exhibits a slow but significant increase in both HRS and LRS cases, suggesting that the voltage of unselected lines is strongly pulled by the input line due to the lack of selectors and thus the abundance of sneak-path currents.

These observations indicate that 1S1R array is a particularly useful technology for SNN applications, which not only improves the output accuracy, but also shields the sneak-path currents and parasitic capacitance on unselected lines, resulting in significantly faster operation and lower power consumption.

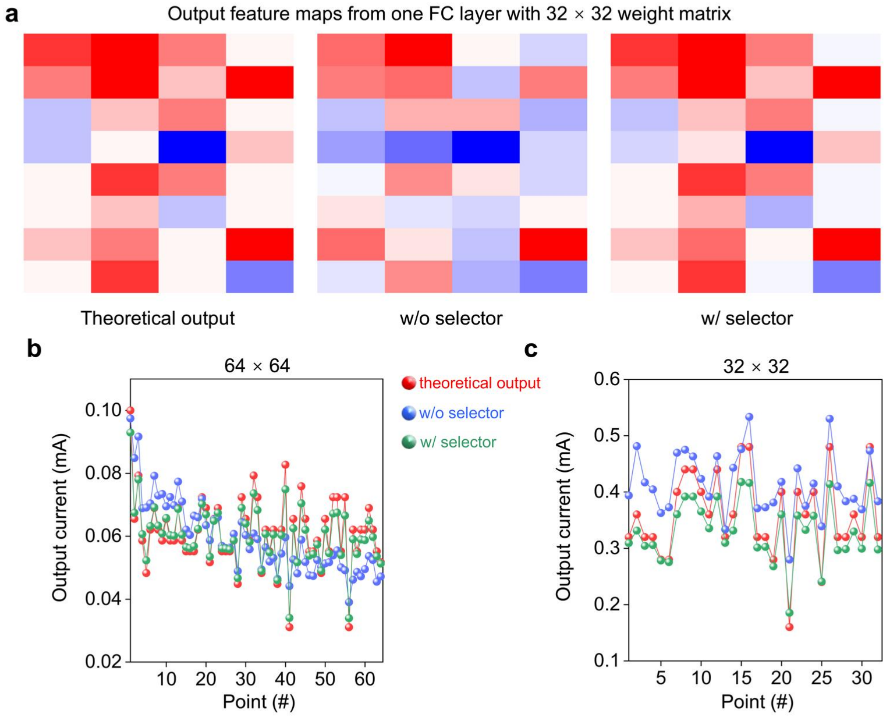

Supplementary Figure 16. **a**, Output feature map obtained by VMM simulation using one fully connected (FC) layer with 32 × 32 weight matrix for the theoretical output (left, normalized to the average output current value), without selector (middle), and with selector (right), respectively. **b,** Output currents of 64 × 64 arrays corresponding to the feature maps. **c,** Output currents of 32 × 32 arrays corresponding to the feature maps.

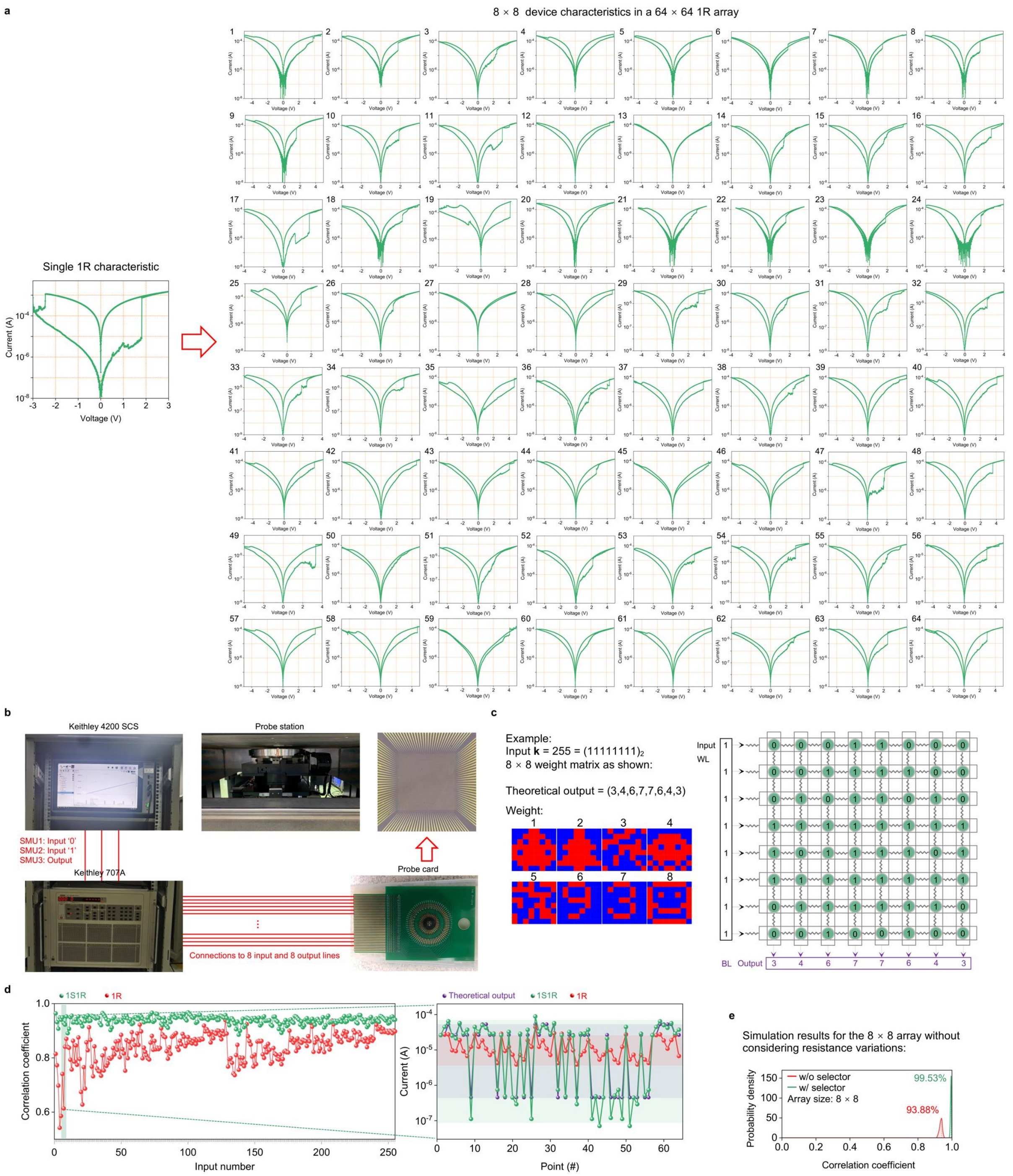

Supplementary Figure 17. Accuracy verification of the subarray in the 64 × 64 array for VMM calculations. a, The operating window of 1R devices tends to decrease as the array size increases due to the presence of sneak-path currents. **b,** Schematic diagram of VMM operation test structure. A probe card is designed to simultaneously probe the 8 input and 8 output lines of the 1R subarray. The 16 input/output channels are connected with the 16 output terminals of a Keithley 707A switch matrix containing two Keithley 7174A low

leakage matrix cards (each with 8 input and 12 output channels). Three channels of the Keithley 707A switch matrix cards are connected to three SMU modules of a Keithley 4200 SCS. Among them, two SMU channels connected to the input lines through the switch matrix, and generates high and low input voltages, respectively. This way, the arbitrary binary input vectors can be generated for the 8 input lines. The other SMU is connected to the output lines through the switch matrix, and can be used to measure the output current while forcing 0 V voltage on a selected output line. **c,** Schematic diagram of the cross-point array performing VMM. **d,** Distribution of correlation coefficients (left) between the output results obtained using 8-bit binary from 1 to 255 as input and the theoretical output results, and the distribution of 64 output currents (right) obtained after using 8 weight settings for one of the 255 groups. 8 sets of 8 × 8 binary weight matrices are generated and programmed into the 8 × 8 array one by one. For each weight matrix, all 2^8=256 possible inputs are applied using the switch matrix and the output current vectors for each input are measured. In order to evaluate the output accuracy, we concatenate the output vectors of each weight matrix to make a 64-long output vector for each input. Due to the yield issue of the 1S1R array, similar experiments are performed with the 4 × 4 1S1R subarray. Each 8 × 8 weight matrix is partitioned into four 4 × 4 weight matrices and the output vectors are measured four times. After that, we concatenate the outputs to obtain the same output format as 1R array. The VMM accuracy can be evaluated by calculating the correlation coefficients of each output vector versus the theoretical output:

$$CorrCoef=\frac{cov(I_{measure,\mathbf{k}},I_{theory})}{\sqrt{var(I_{measure,\mathbf{k}})\times var(I_{theory})}}$$

in which, $I_{\text{measure},\mathbf{k}}$ (**k**=1,…,255) is the measured output current vector for each input **k**, I_{theory} is the theoretical output calculated by computer. The correlation coefficient of the 8 × 8 1R array output results with the theoretical output is 84.15% on average, which improves to 93.93% on average for the 4 × 4 1S1R. **e,** The probability density of the correlation

coefficients of the 8 × 8 array obtained by the same simulation method as the Fig. 3h. It is worth noting that during the theoretical simulations, the HRS and LRS resistances in 1R and 1S1R arrays are set to be constant values (average value of resistance in HRS/LRS), thus the accuracy obtained from the calculation is higher than that of the experiment. From the experimental results we can see that the HRS and LRS resistance variations can also contribute to degradation of accuracies, especially in the case of 64 × 64 1R array. Based on these results, we may conclude that 1S1R allows for a high accuracy way of performing VMM operation.

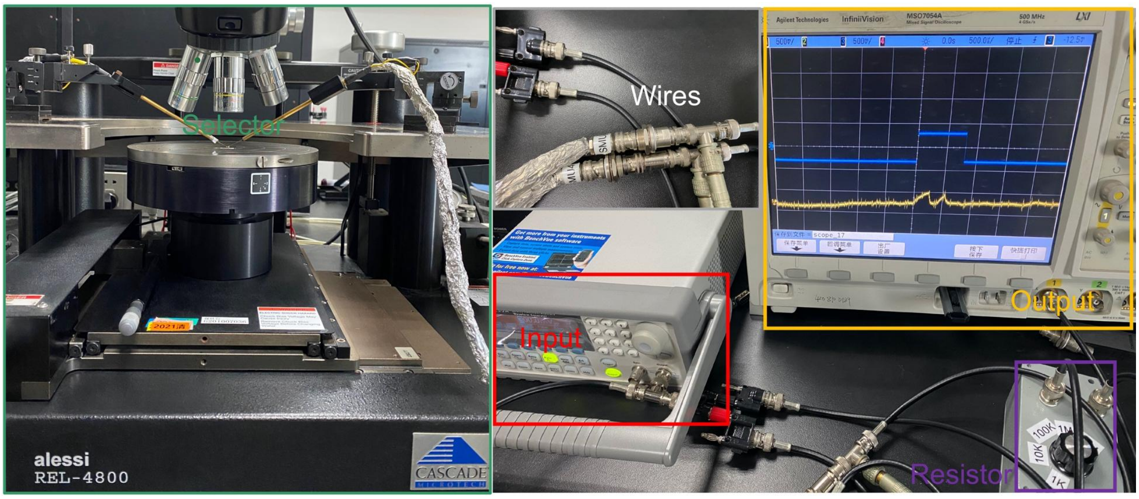

Supplementary Figure 18. Experimental setup for the waveform measurements of selector-based LIF neuron, including a probe station, an Agilent oscilloscope, a Keysight source meter and other components/wiring.

Supplementary References

1. Elton, L. & Jackson, D. F. X-ray diffraction and the Bragg law. *Am. J. Phys.* **34**, 1036–1038 (1966).
2. Holzwarth, U. & Gibson, N. The Scherrer equation versus the'Debye-Scherrer equation'. *Nat. Nanotechnol.* **6**, 534–534 (2011).
3. Wang, Z. *et al.* Memristors with diffusive dynamics as synaptic emulators for neuromorphic computing. *Nat. Mater.* **16**, 101–108 (2017).
4. Guzman, D. M., Onofrio, N. & Strachan, A. Stability and migration of small copper clusters in amorphous dielectrics. *J. Appl. Phys.* **117**, 195702 (2015).
5. Blöchl, P. E. Projector augmented-wave method. *Phys. Rev. B* **50**, 17953–17979 (1994).
6. Kresse, G. & Joubert, D. From ultrasoft pseudopotentials to the projector augmented-wave method. *Phys. Rev. B* **59**, 1758–1775 (1999).
7. Perdew, J. P., Burke, K. & Ernzerhof, M. Generalized gradient approximation made simple. *Phys. Rev. Lett.* **77**, 3865–3868 (1996).
8. Kresse, G. & Hafner, J. Ab initio molecular dynamics for liquid metals. *Phys. Rev. B* **47**, 558–561 (1993).
9. Kresse, G. & Hafner, J. Ab initio molecular-dynamics simulation of the liquid-metal–amorphous-semiconductor transition in germanium. *Phys. Rev. B* **49**, 14251–14269 (1994).
10. Kresse, G. & Furthmüller, J. Efficiency of ab-initio total energy calculations for metals and semiconductors using a plane-wave basis set. *Comput. Mater. Sci.* **6**, 15–50 (1996).
11. Kresse, G. & Furthmüller, J. Efficient iterative schemes for ab initio total-energy calculations using a plane-wave basis set. *Phys. Rev. B* **54**, 11169–11186 (1996).

12. Sheppard, D., Xiao, P., Chemelewski, W., Johnson, D. D. & Henkelman, G. A generalized solid-state nudged elastic band method. *J. Chem. Phys.* **136**, 074103 (2012).

13. Henkelman, G., Uberuaga, B. P. & Jónsson, H. A climbing image nudged elastic band method for finding saddle points and minimum energy paths. *J. Chem. Phys.* **113**, 9901–9904 (2000).

14. Henkelman, G. & Jónsson, H. Improved tangent estimate in the nudged elastic band method for finding minimum energy paths and saddle points. *J. Chem. Phys.* **113**, 9978–9985 (2000).

15. Xue, K.-H. *et al.* Theoretical investigation of the Ag filament morphology in conductive bridge random access memories. *J. Appl. Phys.* **124**, 152125 (2018).

16. Liu, Q. *et al.* Controllable growth of nanoscale conductive filaments in solid-electrolyte-based ReRAM by using a metal nanocrystal covered bottom electrode. *ACS Nano* **4**, 6162–6168 (2010).

17. Barshilia, H. C. & Rajam, K. Raman spectroscopy studies on the thermal stability of TiN, CrN, TiAlN coatings and nanolayered TiN/CrN, TiAlN/CrN multilayer coatings. *J. Mater. Res.* **19**, 3196–3205 (2004).

18. Yang, X. *et al.* Dual-function electron-conductive, hole-blocking titanium nitride contacts for efficient silicon solar cells. *Joule* **3**, 1314–1327 (2019).

19. Cheng, Y., Tay, B., Lau, S. P., Kupfer, H. & Richter, F. Substrate bias dependence of Raman spectra for TiN films deposited by filtered cathodic vacuum arc. *J. Appl. Phys.* **92**, 1845–1849 (2002).
